# GRAPES: A Software for Parallel Searching on Biological Graphs Targeting Multi-Core Architectures

**DOI:** 10.1371/journal.pone.0076911

**Published:** 2013-10-22

**Authors:** Rosalba Giugno, Vincenzo Bonnici, Nicola Bombieri, Alfredo Pulvirenti, Alfredo Ferro, Dennis Shasha

**Affiliations:** 1 Department Clinical and Molecular Biomedicine, University of Catania, Catania, Italy; 2 Department Computer Science, University of Verona, Verona, Italy; 3 Courant Institute of Mathematical Sciences, New York University, New York, New York, United States of America; UGent/VIB, Belgium

## Abstract

Biological applications, from genomics to ecology, deal with graphs that represents the structure of interactions. Analyzing such data requires searching for subgraphs in collections of graphs. This task is computationally expensive. Even though multicore architectures, from commodity computers to more advanced symmetric multiprocessing (SMP), offer scalable computing power, currently published software implementations for indexing and graph matching are fundamentally sequential. As a consequence, such software implementations (i) do not fully exploit available parallel computing power and (ii) they do not scale with respect to the size of graphs in the database. We present GRAPES, software for parallel searching on databases of large biological graphs. GRAPES implements a parallel version of well-established graph searching algorithms, and introduces new strategies which naturally lead to a faster parallel searching system especially for large graphs. GRAPES decomposes graphs into subcomponents that can be efficiently searched in parallel. We show the performance of GRAPES on representative biological datasets containing antiviral chemical compounds, DNA, RNA, proteins, protein contact maps and protein interactions networks.

## Introduction

Biological sequences will always play an important role in biology, because they provide the representation of a fundamental level of biological variability and constitute “evolution’s milestones” [1]. However, technological advances have led to the inference and validation of structured interaction networks involving genes, drugs, proteins and even species. An important task in cheminformatics, pharmacogenomics and bioinformatics is to deal with such structured network data. A core job behind complex analysis is to find all the occurrences of given substructures in large collections of data. This is required for example in (i) network querying [2]–[5] to find structural motifs and to establish their functional relevance or their conservation among species, (ii) in drug analysis to find novel bioactive chemical compounds [6], [7], and (iii) in understanding protein dynamics to identify and querying structural classification of protein complexes [8].

The networks consist of vertices as basic elements (i.e., atoms, genes, and so on) and edges describe their relationships. All cited applications build on the basic problem of searching a database of graphs for a particular subgraph.

Formally, graph database searching is defined as follows. Let 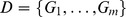 be a database of connected graphs. A graph 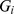 is a triple 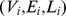. 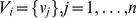 is the set of vertices in 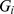. 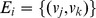, 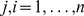, is the set of edges connecting vertices in 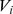. We consider edges to be undirected. The degree of a vertex 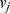 is the number of edges connected to it. Each vertex may have a label, representing information from the application domain.

**Figure 1:**
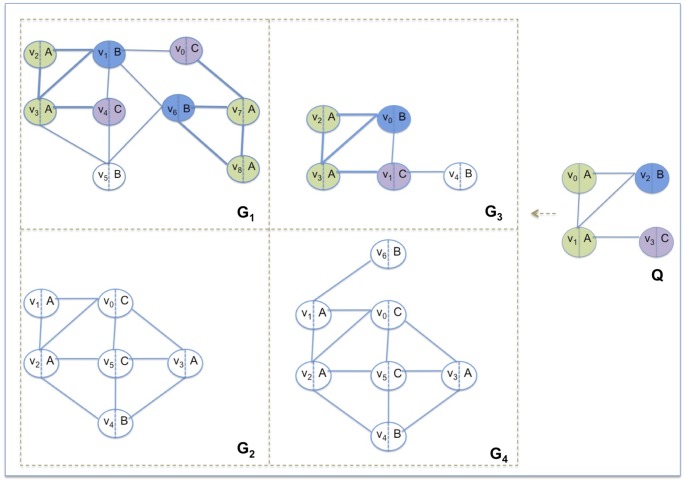
Graph database and query. The database is composed by four graphs 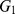, 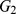, 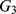 and 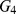. 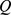 is the query. The occurrences of 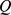 in the graphs are shown by colored vertices and bold edges.

Let 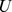 be the set of all possible labels. Let 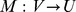 be the function that maps vertices to labels. Let 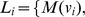 for all 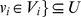 be the set of labels of 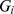. For each graph in the database, each vertex has a unique identifier, but different vertices may have the same label. Figure 1 shows an example of a database of graphs and a query. In this case 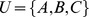 coincides with 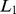, 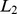, 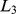 and 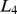. Examples of mapping may be 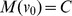 in 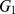 and 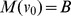 in 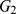.

Two graphs 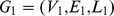 and 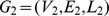 are *isomorphic* if and only if there exists a bijective function 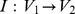 mapping each vertex of 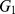 to a vertex of 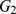 such that 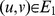 if and only if 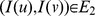 and vice versa. We must respect also the *compatibility* of the labels of each mapped items, such that 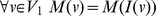.

A *subgraph isomorphism* (hereafter, also called subgraph matching or matching) of 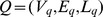 in 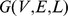 is an injective function 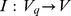 such that 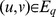 if and only if 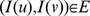 and 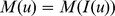 and 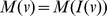. Note that, there may be an edge 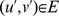 without any corresponding edge in 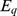. Given a set of graphs 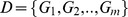 and a graph 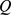, called query, the problem consists of identifying the graphs in 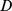 containing 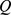 as a subgraph together with all the locations, called occurrences, of 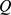 in those graphs. This problem is called *graph sub-isomorphism* and the complexity of all existing exact approaches is exponential. In Figure 1, colored vertices and thick edges highlight the subgraph isomorphisms of 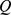 in the set of graphs.

Much research has been done to try to reduce the search space by filtering away those graphs that do not contain the query. This is achieved by indexing the graphs in 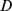 in order to reduce the required number of subgraph isomorphism tests. Because graphs are queried much more often than they change, indexes are constructed once by extracting structural features of graphs in a preprocessing phase. Features are then stored in a global index. Later, given a query graph, the query features are computed and matched against those stored in the index [9]. Graphs having the features of the query are *candidate* to contain the query. The set of candidates is then examined by a subgraph isomorphism algorithm and all the resulting matches are reported. The time spent searching on these graphs is exponential in the graph size. Heuristic (sub)graph-to-graph matching techniques [10], [11] are used to solve this exponential step. Other tools based on the identification of discriminant characteristics of graphs [12], [13] are used.

Almost all solutions build the index on subgraphs (i.e., paths [14]–[16], trees [17], [18], graphs [19]) of size not larger than ten vertices to save on time and space.

The memory footprint and the time spent for building the index may be prohibitive even when applied to small subgraphs encouraging the use of compression heuristics. SING [15] stores paths of vertex identifiers in its index as well as the label of the first node in each path. GraphGrepSX [16] stores the paths in an efficient data structure called trie which compacts common parts of features.

Other systems use data mining techniques, which have been applied to store only non-redundant frequent subgraphs (i.e., a subgraph is redundant when it filters as much as its subgraphs) [18]–[20]. GraphFind [21] uses paths instead of subgraphs but reduces the number of indexed paths by using low-support data mining techniques such as Min-Hashing [22]. In spite of these heuristic techniques, index construction remains an expensive step.

The topology of the features affects both construction time and query effectiveness. TreePi [17] pioneered the use of trees as features. The authors describe a linear-time algorithm for computing the canonical labeling of a tree. They experimentally show that tree features capture topological structures well. Therefore, using trees may result in a good compromise between construction efficiency of query filtering effectiveness. As shown by the authors, a unique center can be defined for a tree. Consequently the distance (shortest path) between pairs of features in a graph can be computed. TreePi uses an additional pruning rule based on distances between features to improve the quality of the match. Tree+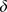 [23] uses as features both trees and a restricted class of small graphs to improve the filtering performance. A most recent contribution in the literature which uses trees is CT-index [24]. In particular it hashes the indexed features which are a combination of trees and cycles. Trees and cycles are represented by their canonical form and mapped into the fingerprint by the hashing function. The loss of information caused by the use of hash-key fingerprints seems to be justifiable by the compact size of the indexing as long as the amount of false positive does not increase significantly due to collisions. The matching algorithm is driven by a fixed ordering of the query vertices. The vertex sequence is built looking for connectivity and using a priority based on label frequencies respect to the whole graphs database. Unfortunately, as for graphs, enumerating all trees of bounded size still produces a large number of features.

Therefore, while the above approaches have good performance on medium-small size graphs, they are not suitable when the size and density of graphs increase. Some approaches have been proposed to deal with very large graphs [25]. In such cases, the massive data sets are decomposed and distributed onto cluster-based architectures. Nevertheless, the time overhead of the distributed computational model is high compared to the overhead on a symmetric multiprocessor (SMP) architecture.

In any parallel setting, multiple instances of the state of the art searching software can be run on the CPU cores, each one on a disjoint database graphs. These do not work when the database consists of a single large graph (as is often the case with biological networks). In addition, the parallel search may be imbalanced even on databases of many graphs, due to the differences among graph sizes and densities.

This paper presents GRAPES, a parallel algorithm for biological graph searching on multicore architectures. GRAPES is a parallelized version of the index-based sequential searching algorithms proposed in [15], [16]. It introduces new algorithmic strategies tailored to parallelism. The main characteristics of GRAPES when compared to [15], [16] in Table 1, are the following:

**Table 1:** GRAPES vs GraphGrepSX and SING.

	GRAPES	GraphGrepSX	SING	Description
TRIE indexing	x	x		Compact indexing. Efficiency on indexing and filtering
	x	x		
Path indexing	x	x	x	Lighter than subgraphs or trees
Store path node	x		x	More filtering power
Parallel Indexingand Matching	x			Necessary for biological data size and density
Connected components	x			Load balancing onto CPU cores

List of the new GRAPES components together with the components of GRAPES taken from GraphGrepSX [16] and SING [15].

- Each single graph of the database is indexed in parallel by 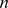 threads, where 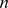 is the number of processing units. Each index construction is independent and dynamically distributed to the threads. This guarantees load balancing and minimal synchronization overhead, regardless of the number, size and density of the database graphs.
- GRAPES uses paths of bounded length as features, and *Trie* structures to represent them. Path prefix sharing in tries reduces data redundancy thus achieving a more compact representation of the index. The index also stores the number of occurrences of the features and their locations.
- GRAPES implements a filtering phase, which consists of the *trie* comparison of all query graph features against the pre-computed global *trie* of the database.
- The matching phase applies the well established graph isomorphism algorithm proposed in [10], with the exception that only suitable subgraphs within candidate graphs (i.e., that pass the filtering phase) are analyzed. This runs in parallel both within each subgraph and among subgraphs thanks to the indexes built in the first phase.

The experimental results show that GRAPES efficiently exploits the computing power of the multicore architectures by guaranteeing scalability and load balancing. The experimental results also show that GRAPES provides better performance than sequential solutions run in parallel on databases of graphs, and provides fast searching results even on databases of graphs that would be intractable by applying sequential searching algorithms.

The paper is organized as follows. Section presents GRAPES in detail. Section presents the experimental results while Section is devoted to concluding remarks.

## Methods

### Software Overview

**Figure 2:**
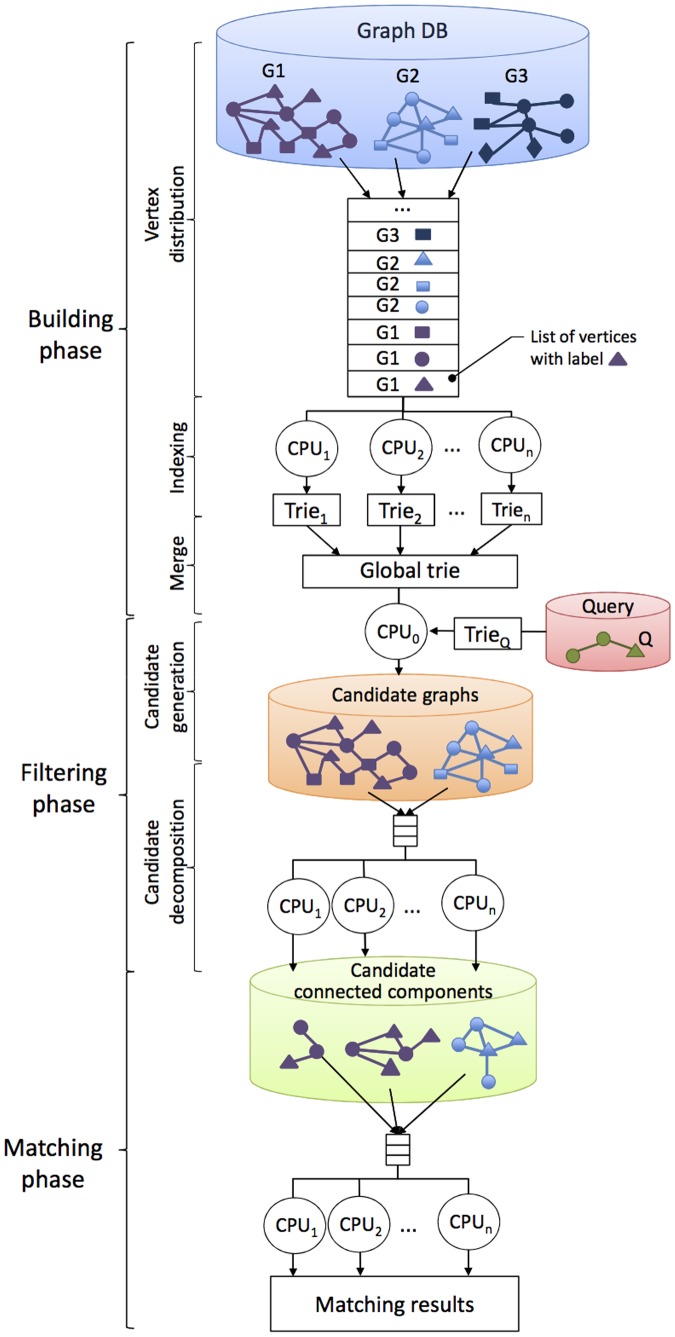
Schema of GRAPES.

GRAPES implements a parallel search algorithm for databases of large graphs targeting multicore architectures. It consists of the following three phases (see Figure 2):

- *Building phase*. Given a database of graphs, the building phase constructs an index called a *global trie*. The global trie consists of the set of substructures (paths of length up to a fixed value called 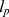), hereafter called *features*, of the database graphs, as described in Section.
- *Filtering phase*. Given a query, GRAPES builds an index of the query (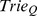) and compares that index to the features of the graphs. It discards graphs that do not contain the query features. In the remaining graphs, which are called *candidate graphs*, it filters those parts (subgraphs) that are not present in the query graph. This step may disconnect the candidate graphs by decomposing them into a set of connected components, hereafter called *candidate connected components*, as described in Section.
- *Matching phase*. A subgraph matching algorithm (i.e., [10]) is run to find all the occurrences of the query in each connected component in parallel, as described in Section.

### Building Phase: Extracts the Features and Constructs a Trie Index

This phase builds a trie index of the whole database. The index is built in parallel by the 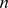 threads run on the CPU cores. For each graph, GRAPES groups the vertices in lists, all the vertices in the same list have the same label (see Figure 2). The lists are dynamically distributed to the threads. Each thread instantiates a partial index (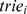), which stores all feature, i.e. all paths of labels, information starting from the vertices of the assigned list. Each partial index is built independently and asynchronously. A thread that collects all the information of the assigned list proceeds with a further list related to the same graph or even to another graph. This guarantees load balancing on the CPU cores regardless of the number, size, and density of the database graphs.

**Figure 3:**
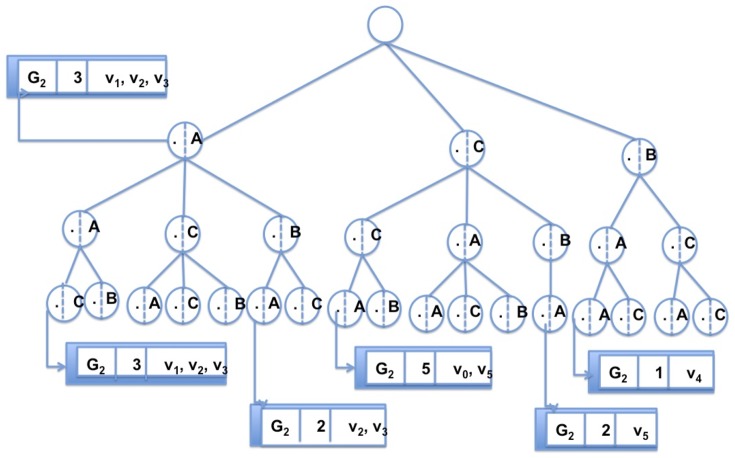
The trie data structure used as indexing in GRAPES. The index for the graph 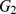 of Figure 1. A trie stores all features of length 2 of 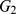. Each node is associated to a feature, that is the path in the trie up to this node. For each node in the trie, a list of information such as the number of times the feature appears in 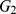 (equally the number of paths in 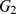 mapped to it) and the identifications of the first nodes of such paths is stored.

For each vertex 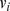, the thread stores all label paths starting from 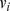, 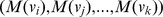 containing up to 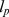 vertices. Recalling that different vertices may have the same label, the thread records the number of time each feature (which is a topological pattern, here a sequence, of labels) appears in the graph and the identification (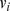) of the first vertex of the paths. For example, the feature (C,C,A) of length two in graph 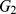 of Figure 1 occurs 5 times (see Figure 3 to locate the feature indexed in the trie). The paths mapped to it are 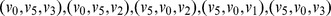. The identifications of the first vertices of such paths are 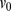 and 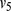.

After it completes its collection phase, each thread stores all label path features in the partial trie. Each node of the trie tree of height 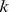 is a path of labels 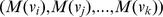, of length 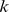. The height of the trie is 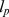. All the descendants of a node share a common prefix of the feature associated with that node. Each node in the trie links to (i) the list of graphs 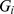 containing the feature associated with that node, (ii) the number of times the feature occurs in each 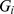 (that is the number of paths 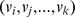 mapped to 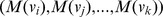 in each 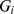), and (iii) the starting vertices 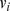 of each such path.

**Figure 4:**
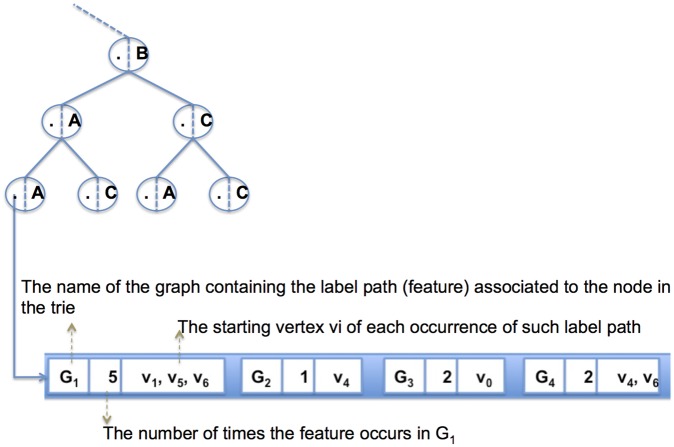
The information stored in a node of a GRAPES index (trie) for graphs of Figure 1. Each node in the trie links to (i) the list of graphs Gi containing the feature associated with that node, (ii) the number of times the feature occurs in each Gi, and (iii) the starting vertices vi of each such path.

Then, all the tries partially built by each thread are merged into the final global trie (this is done sequentially since it is very short and fast). For example, Figure 3 depicts the global trie of a database composed of a single graph. Let’s 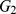 of Figure 1 be such a graph. The features are paths of length two (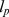 = 2). Note that the root is virtual (does not correspond to any vertex). All paths in the trie (starting from nodes at level 1 to the leaves) are features in 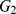. All nodes link to a list of information as described before (the figure shows the result for only some nodes). Figure 4 depicts the list of information for the third node in path (B,A,A) for all graphs in Figure 1.

### Filtering Phase: Reduce the Search Space

The filtering phase consists of two steps: (i) filter away those graphs that cannot have a match with the query graph (i.e., candidate generation) and (ii) filter away the portions of potentially matching graphs that have no chance of matching the query graph (i.e., candidate decomposition into connected components).

**Figure 5:**
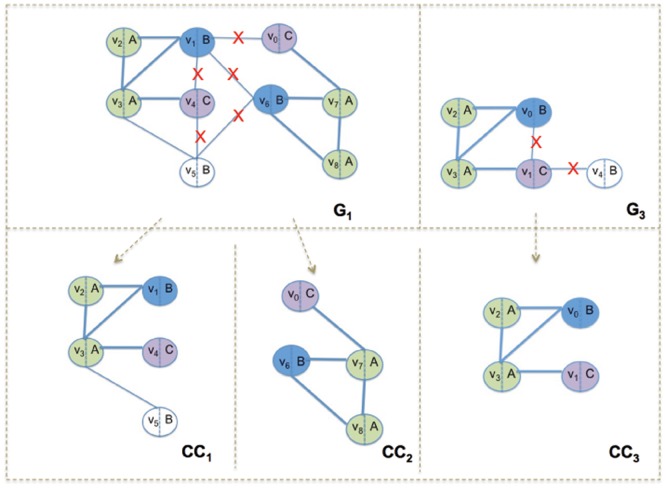
Filtering in GRAPES. The graphs in Figure 1 are reduced to the following components after filtering. 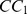 and 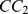 derive from 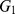 and 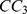 from 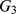. This reduction of search space makes GRAPES fast and suitable to parallelization. The combinatorial subgraph isomorphism algorithm (applied in the Matching phase) will be run only on these components.

1. Given a query *Q*, GRAPES extracts all features from *Q*. Note that features in *Q* are paths up to length 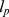, the same length value used to construct the index for the graphs of the databases. All features are stored in a trie (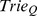).
2. GRAPES matches the trie of the query *Q* against the *global trie*. The algorithm discards those graphs that either have a feature with an occurrence number less than the occurrence number of the query or do not contain some features of the query graph. For example in Figure 1, 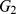 is discarded because the feature (B,A,A) appears once in 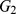 and twice in the query.
3. For each vertex *u* of the query graph *Q* and a potentially matching vertex 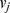 in graph 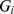, any feature starting from *u* should also start from 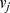. Otherwise 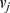 cannot be a match. GRAPES achieves this by looking at the tries of the starting vertices 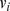 of the occurrences of features in the graphs. Thus, for the paths in the trie of *Q* matching paths in the trie of *D*, and for all of those starting from the same vertex *u* in *Q*, all occurrences of the matching features in those graphs 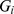 must also start from the same vertex 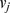. Otherwise, 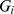 cannot match *Q* starting at 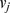. Figure 1 depicts such a case, in which 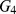 contains (B,A,A) twice as does the query. However, in the query the paths start from a single node, i.e. 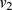, whereas, in 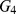, the two paths start from two vertices, i.e. 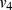 and 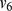.
4. GRAPES retrieves from the trie the identities of the starting vertices of the paths in the data graphs that match the query features. The net effect is that GRAPES extracts all maximally connected components in the graphs involving only possible vertices. This is done in parallel by dynamically assigning each candidate graph to threads for guaranteeing load balancing. For example, when searching for *Q* in the database of Figure 1, GRAPES reduces the search space by returning only the connected components depicted in Figure 5.

This approach is particularly helpful when the graphs in the database are very large. Because the filtering step decomposes the graphs with respect to the query, large graphs become a set of smaller components, greatly reducing the load on the exponential matching phase as well as enabling that phase to be parallelized. Parallel operation (for index creation, index searching, and heuristic subgraph isomorphism) and the decomposition of graphs into small connected components are the main reasons GRAPES is so fast.

### Matching Phase: Finds All Occurrences of the Query in the Graphs in the Database

GRAPES runs multiple instances (i.e., one for each thread) of the combinatorial subgraph isomorphism algorithm *VF2* [10] on the connected components resulting from the filtering phase.

*VF2* is a combinatorial search algorithm which induces a search tree by branching states. It uses a set of topological feasibility rules and a semantic feasibility rule, based on label comparison, to prune the search space. At each state if any rule fails, the algorithm backtracks to the previous step of the match computation.

The size and the density of the generated connected components may be different. As a consequence, distributing each connected component as a whole for matching to each thread may cause load imbalance. On the other hand, many instances of the matching algorithm on the same connected component likely lead to redundant visits in the same search space.

GRAPES applies a heuristic to select (i) the number of searching instances to be run in parallel on a single connected component at a time, and (ii) the starting nodes for the searching instances of each connected component. The heuristic relies on the lists of *matchable vertices* that have been extracted for each vertex of the query during the filtering phase (see step 3 of Section). There is one list of matchable vertices per connected component. Longer lists are processed first, because one instance of the matching algorithm can be run in parallel starting from each node of the list.

### Availability and Requirements

Project name: GRAPES

Project home page: http://ferrolab.dmi.unict.it/grapes.html, https://code.google.com/p/grapessmp

Operating system(s): Unix/Linux x86/x86-64

Programming language: C++

Other requirements: GRAPES runs in parallel on shared memory multi-core architectures

License: GRAPES is distributed under the MIT license.

## Results and Discussion

GRAPES has been tested on four databases of biological graphs:

- *AIDS*, the standard database of the Antiviral Screen Dataset [26] of the topological structures of 40,000 molecules. Graphs in the dataset have 62 unique labels in total. The average number of vertices per graph is 44.98 with a standard deviation of 21.68. The average (resp. standard deviation) degree per vertex is 4.17 (resp. 2.28) and the average (resp. standard deviation) number of labels is 4.36 (resp. 0.86). They are small sparse graphs whose maximum size is 245.
- *PDBS*, a database of 600 graphs representing DNA, RNA and proteins having up to 16,431 vertices and 33,562 edges [27]. The dataset has been converted to graphs by using the Ball conversion library available at www.ball-project.org/; original structures can be downloaded from www.fli-leibniz.de/ImgLibPDB/pages/entry_list-all.html. As reported in [27], the PDBS dataset contains echoviruses and decay-accelerating factor (DAF). The dataset contains a total of 10 unique labels with an average of 6.37 and a standard deviation of 2.15 per protein. The average degree per vertex is 4.27 with a standard deviation of 2.02. The average number of vertices (resp. edges) is 2,939 (resp. 6,127) with a standard deviation of 3,214 (resp. 6,522).
- *PCM*, the protein contact maps dataset is taken from *CMView* [28]. It comes from 200 contact maps of the amino acids of the domains of the proteins having up to 883 nodes and 18,832 edges. Since they represent relationships among amino acids, the number of vertices in the contact maps is relatively small (corresponding to the length of the proteins). The average number of vertices (resp. edges) is 376 (resp. 8,679) with a standard deviation of 186.6 (resp. 3,814). The average degree per vertex is 44.78 with a standard deviation of 17.47. The average number of labels per contact map and the standard deviation are 18.86 and 3.48, respectively.
- *PPI*, a database of 20 protein interaction networks. The networks belong to 5 species: *Caenorhabditis elegans, Drosophila melanogaster, Mus musculus, Saccaromyces cerevisiae* and *Homo sapiens*. For each species, we generated four networks from the original ones, by selecting the edges having accurateness greater than 0.4, 0.5, 0.6, and 0.7. The networks are annotated using Gene Ontology annotations [29]. We run the Cytoscape plugin Mosaic [30], which assigns colors to the vertices of the networks depending on their most relevant GO terms. The networks have up to 10,186 nodes (average 4,942.9 and standard deviation 2,648) and 179,348 edges (average 53,333 and standard deviation 51,387). The average degree per vertex is 18.46 with a standard deviation of 42.12. The average number of labels per graph and the standard deviation are 28.45 and 9.5, respectively.

The queries for the *AIDS* database are entire compounds randomly chosen from the database of size 8, 16 and 32 edges. For the datasets *PDBS* and *PCM*, the queries have the same size but they have been generated from the original graphs in the following way. Starting from an edge of the graph, choose a random neighboring edge. In general, choose any edge that neighbors any edge of the growing graph. The process is repeated until the chosen number of edges is reached. Since queries are substructures chosen randomly from the databases, the queries reflect the average degree and label distribution of the biological data. For the *PPI* dataset, since relevant queries are often small graphs (e.g., feed forward loops), we used queries of size 4 and 8. Results are given as the average performance obtained by running from one to one hundred queries.

Experiments have been conducted on a SMP machine 6×2.8 GHz Intel Xeon with Debian 5.0×86 O.S. GRAPES has been implemented in C++ and compiled with gcc-4.4.

We compare GRAPES with available software implementing sequential graph searching algorithms based on indexing such as SING [15], GraphGrepSX (GGSX) [16], and the most recent CT-index [24]. We also report the results obtained by running *VF2* [10], which does not use indexing. We do not report comparisons with gIndex [19], GCoding [13] and CTree [12] since SING either outperforms them or gives closely comparable performance (we refer to [15] for details of such comparisons). The results (building time, matching time and building+matching time) are presented as the average time of 100 queries, run one at the time. For each query, we set the system to wait 8 hours for the searching results. When we state that a tool does not run on a dataset, we mean that the tool did not terminate (within 8 hours) in almost all tested queries. SING runs out of time on *PDBS*, *PCM* and *PPI*, while CT-index runs out of time on *PCM* and *PPI*.

**Figure 6:**
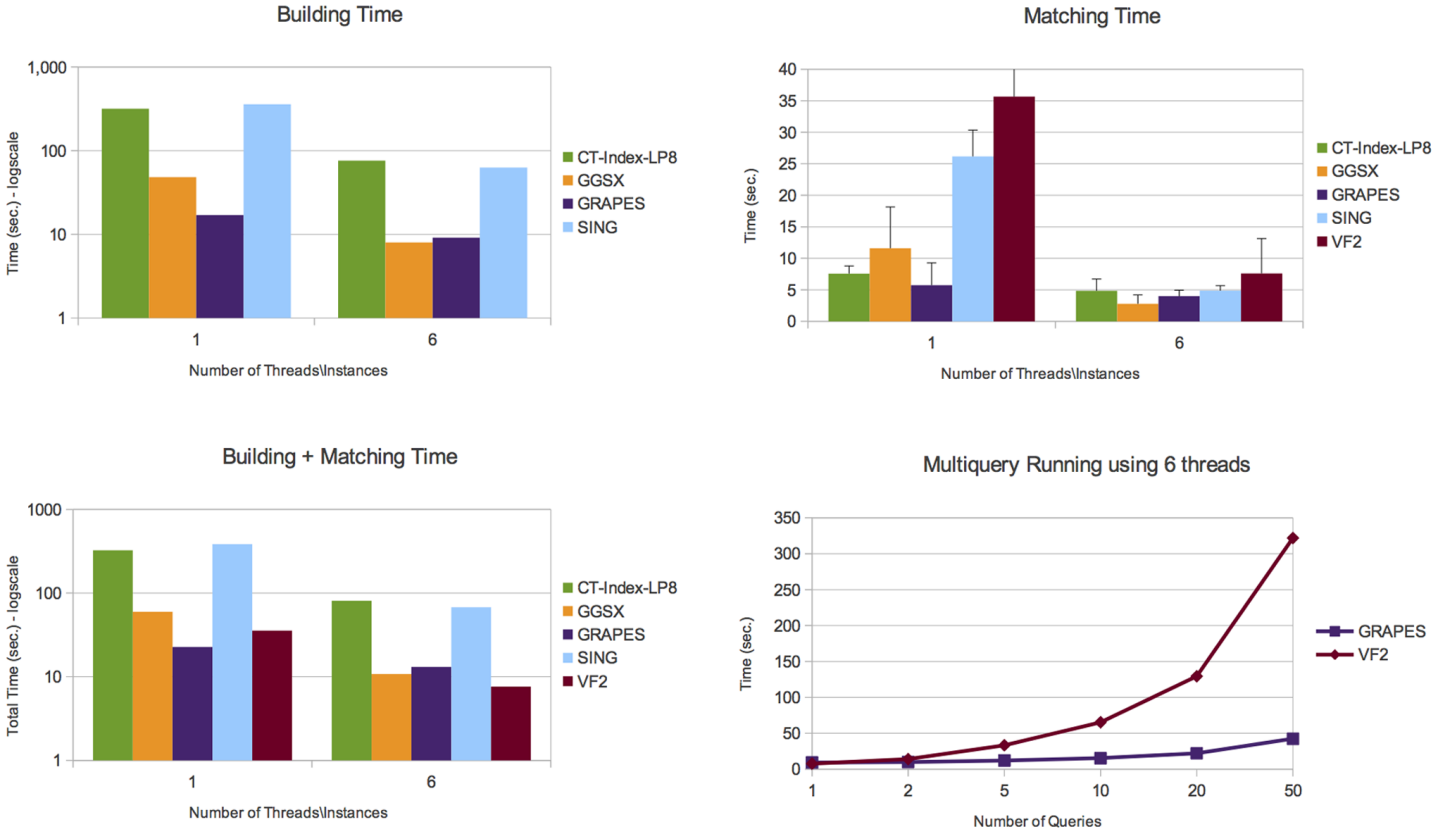
Experimental results on the *AIDS* database. It shows building, matching and total (building+macthing) time of the compared tools. The *AIDS* dataset contains a large number of small, simply structured graphs. Here, querying is not an expensive task. Comparisons show that GRAPES outperforms the other tools by using 1 thread. Parallelism due to the simple structure of data does not help. However, indexing induces efficiency when the index is built once and re-used for multiquery. Indeed, GRAPES results faster than non-indexing based methods such as VF2 by running two o more queries (see Multiquery running plot, run on 6 threads).

**Figure 7:**
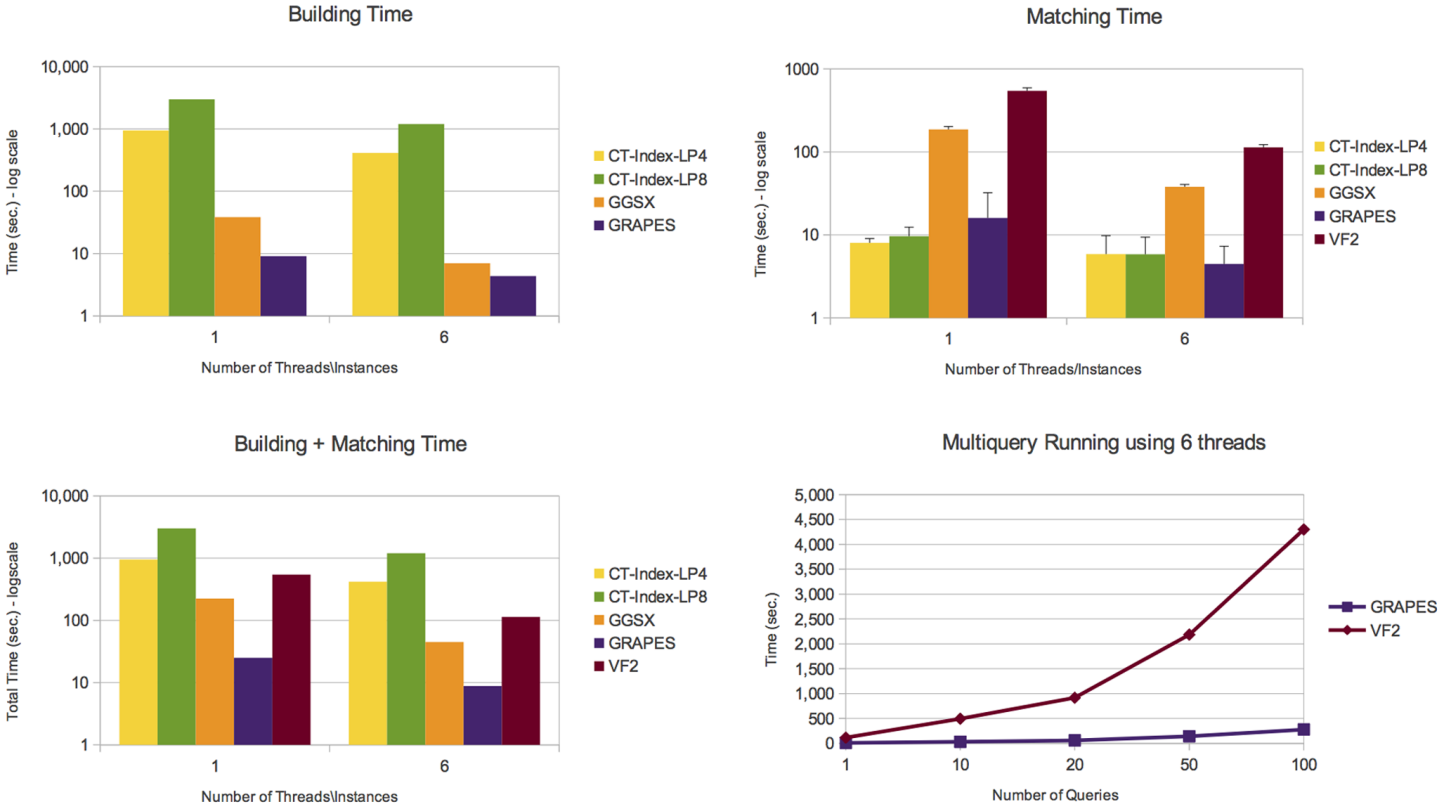
Experimental results on the *PDBS* database. It shows building, matching and total (building+macthing) time of the compared tools. Comparisons show that GRAPES outperforms the other tools by using 1 or 6 threads. However, parallelism helps to decrease the computation costs. We found that indexing induces efficiency when the index is built once and re-used for multiquery. Indeed, GRAPES results faster than non-indexing based methods such as VF2 by running one or more queries (see Multiquery running plot, run on 6 threads).

**Figure 8:**
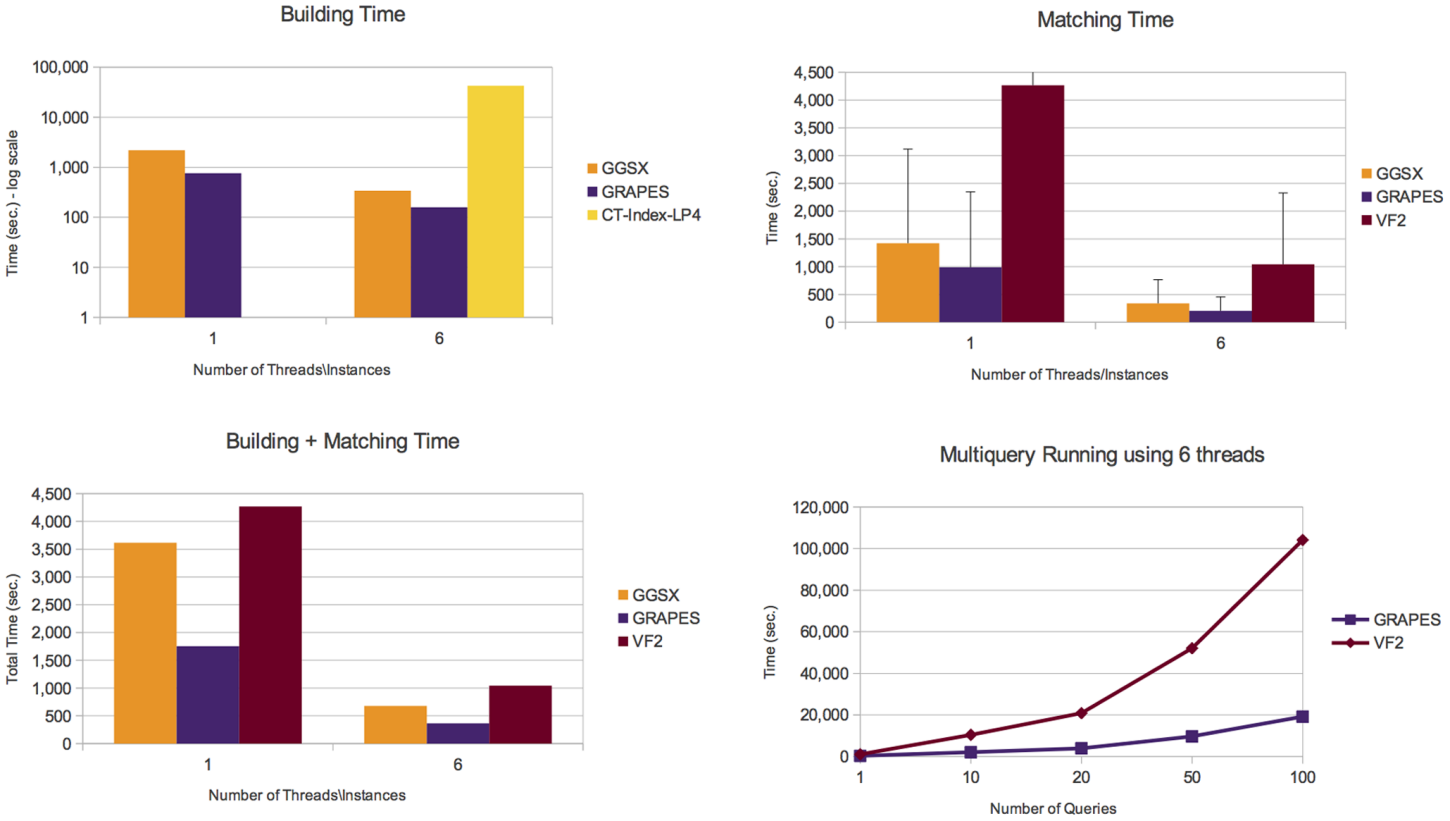
Experimental results on the *PCM* database. It shows building, matching and total (building+macthing) time of the compared tools. Comparisons show that GRAPES outperforms the other tools by using 1 or 6 threads. However, parallelism helps to decrease the computation costs. We found that indexing induces efficiency when the index is built once and re-used for multiquery. Indeed, GRAPES results faster than non-indexing based methods such as VF2 by running one or more queries (see Multiquery running plot, run on 6 threads).

**Figure 9:**
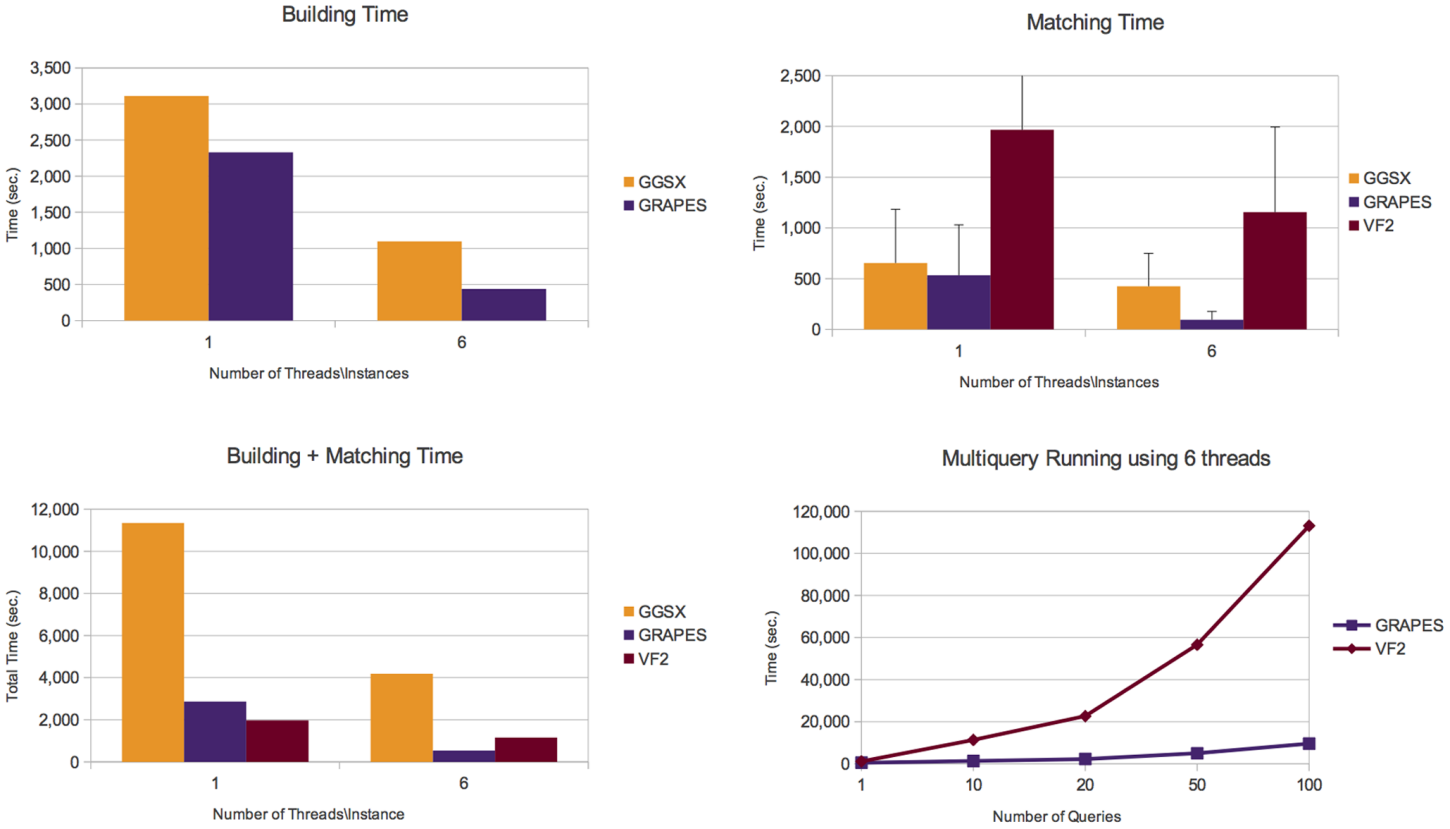
Experimental results on the *PPI* database. It shows building, matching and total (building+macthing) time of the compared tools. Comparisons show that GRAPES outperforms the other tools by using 1 or 6 threads. However, parallelism helps to decrease the computation costs. We found that indexing induces efficiency when the index is built once and re-used for multiquery. Indeed, GRAPES results faster than non-indexing based methods such as VF2 by running one or more queries (see Multiquery running plot, run on 6 threads).

Details on the number of graphs containing the queries, the number of subgraphs isomorphisms and the number of connected components are given in Table S1 in the File S1. Figures 6, 7, 8, and 9 report the obtained results on *AIDS*, *PDBS*, *PCM* and *PPI*, respectively. Each figure reports the number of the building and matching time and the performance of GRAPES and *VF2* for the runs of multiple queries (i.e., from one to one hundred queries). For a fair comparison, we also run multiple instances (six) of the state of the art algorithms on different graphs of the databases.

We report the average time and the corresponding standard deviations obtained on the tested queries. The filtering time, which is negligible for all the tested graphs, is included in the matching time. The number of candidates graphs and memory consumption of the compared tools are reported Figures S1–S4 in the File S1.

On all tested databases (Figures 6, 7, 8, 9), considering the building plus matching time, GRAPES is faster than VF2 even when only one query is run. Therefore, even though indexing is a time-consuming step, it clearly speeds up the matching time. Parallelism is critical to this success. In Figures 6 and 9, when using only one thread, the index-based tools do not outperform VF2 when a single query is run. The index-based tools outperform VF2 when we compare the performance of GRAPES and *VF2* (building plus matching time, see multiquery running graphs in Figures 6, 7, 8, 9) on one hundred queries using six threads for GRAPES and six instances of *VF2* over the databases. These results show that indexing induces efficiency when the index is constructed once and re-used for multiquery. Parallelism and our particular index strategy give substantial improvements over index-less systems such as *VF2*, with increasing benefits the more queries there are.

On the *AIDS* dataset (Figure 6), GRAPES is faster than SING and CT-index and comparable to GGSX. We run CT-Index using the size of features (i.e., 8) suggested by the CT-index authors on the same dataset (LP8 means trees of depth 6 or cycles of 8 edges).

GRAPES has a particular advantage compared to other indexing-based graph searching or subgraph isomorphism algorithms on more complex structured graph databases. For example, on the *PDBS* (Figure 7) and *PCM* (Figure 8) datasets, GRAPES is much faster than GGSX and CT-Index. We run CT-Index using also small size features (LP4), to evaluate a possible matching and building speedup. However, since CT-Index in *PCM* (Figure 8) dataset has a very high building time, GRAPES and GGSX outperformed it in the total time. For this reason, its matching time is not reported in the plot.

GRAPES is faster than GGSX also in *PPI* (Figure 9) dataset. In the *PDBS* (Figure 7), *PCM* (Figure 8), and *PPI* (Figure 9) datasets, GRAPES is much faster than VF2 even on a single query (building plus matching time).

GRAPES is faster than the compared tools in all datasets also when queries are not present in the datasets (see Figures S6–S9 in the File S1).

We conclude that the parallelism of GRAPES yields load balancing, scalability, and efficiency on all databases (see Figure S5 in the File S1). The index-based approach implemented in GRAPES is well-suited to biological data particularly when data has complex structure and queries are time consuming. Further, GRAPES performance is better than multiple instances of sequential graph-search tools of the literature.

## Conclusions

GRAPES is a parallel graph searching algorithm that achieves the best performance of any algorithm on large data coming from applications such as pharmacogenomics and biology. Its advantage is the strongest for large graphs as opposed to databases of many small graphs. GRAPES is based on the following algorithmic strategies:

1. Indexing occurs in parallel on vertices, so there is parallelism even if the entire database consists of a single graph. Further, the indexing for different nodes can operate independently and asynchronously.
2. GRAPES uses the indexing system to partition large graphs into connected components, thus substantially reducing the effort when the exponential phase arises. This makes GRAPES suitable for applications on large biological graphs such as protein interaction networks.
3. To compress the resulting path-based indexes, GRAPES uses tries. In doing so, GRAPES reduces the space and time needed for the construction of large database indexes.

We have demonstrated that GRAPES improves performance compared with state of art algorithms by a factor of up 12, and offers parallel scaling with a factor varying from 1.72 to 5.34 on the biologically representative datasets containing antiviral chemical compounds, DNA, RNA, proteins, protein contact maps and protein interactions networks.

## Supporting Information

File S1 Details on GRAPES performances. It shows for the compared tools memory consumption as well as the number of candidate graphs, connected components found in various setting and the performance of the tools when the queries have no matches.
(PDF)

File S2 GRAPES user manual.
(PDF)
