# Supplementary material for: GRAPES: A Software for Parallel Searching on Biological Graphs Targeting Multi-Core Architectures
Source: PLoS One. 2013 Oct 22;8(10):e76911. doi: 10.1371/journal.pone.0076911 (PMC3805575; doi:10.1371/journal.pone.0076911)
Supplement: File S1 — Details on GRAPES performances. It shows for the compared tools memory consumption as well as the number of candidate graphs, connected components found in various setting and the performance of the tools when the queries have no matches. (PDF) [file pone.0076911.s001.pdf]

Details of GRAPES performance

GRAPES achieves good performance since its indexing, filtering and parallelism work well. This supplement shows memory consumption as well as the number of candidate graphs and connected components found in various setting. Figures S1,S2,S3,S4, and S5 show measurements over the average of 100 queries. Table S1 reports the number of graphs in the datasets containing the queries, the number of subgraph isomorphisms and the number of connected components generated by GRAPES, each of which can be treated in parallel.

The number of candidates graphs obtained after filtering and the number of graphs containing the queries (called matching graphs in Table S1) is useful to understand the effectiveness of the indexing and filtering methods. All tested algorithms give the same number of subgraph isomorphisms.

The *AIDS* database contains 40,000 graphs. On average, 3,469 graphs contain each query at least once (Table S1). Within these 3,469 graphs, each query occurs about 123,839 times (Table S1). On average, GRAPES disconnects 7,472 candidate graphs (Figure S1) into 12,239 connected components, which allow GRAPES parallelism. GRAPES consumes more memory than SING, VF2 and GGSX, but much less than CT-Index (Figure S1). CT-Index filters better and generates fewer candidate graphs than GRAPESĠRAPES generates fewer candidates graphs than SING and GGSX. VF2 does not perform indexing or filtering, therefore the memory it consumes is related only to the matching phase and it is very low.

Concerning the memory, possible improving of GRAPES may valuate the use of only a single trie to index the features and share among threads. However, this strategy should deal with synchronization and related issues. Another way could be to compact the bit vectors used to store the starting vertices of features [**?**].

The *PDBS* database contains 600 graphs. On average 463 graphs contain each query at least once (Table S1). In those 463 graphs, each query occurs about 129,233 times(Table S1). On average GRAPES disconnects 553 candidate graphs (Figure S2) into 7,913 connected components allowing GRAPES parallelism. GRAPES again consumes more memory than SING, VF2 and GGSX, but less than CT-Index (Figure S2). In this case, GRAPES filters best, generating the fewest candidate graphs of any of the methods.

The *PCM* database contains 200 graphs. On average, 101 graphs contain each query at least once (Table S1). In the 101 graphs, each query occurs about 134,306,322 times(Table S1). On average, GRAPES disconnects the 101 candidate graphs (Figure S3) into 120 connected components allowing GRAPES parallelism. GRAPES consumes more memory than VF2 and GGSX (Figure S3), but filters better.

The *PPI* database contains 20 graphs. On average 12 graphs contain each query at least once (Table S1).

	AIDS	*PDBS*	*PCM*	*PPI*
Matching<br>Graphs	3,469<br>(983)	463<br>(61)	101<br>(3.86)	12<br>(2)
Subgraph<br>Isomorphisms	123,839<br>(50,869)	129,233<br>(121,198)	134,306,322<br>(157,648,779)	52,782,349<br>(18,967,658)
GRAPES Connected<br>Components	12,239<br>(10,952)	7,913<br>(2,922)	120<br>(5.87)	12.50<br>(2.03)

Table S1. Subgraph isomorphisms information

In the 12 graphs, each query occurs about 52,782,349 times (Table S1). On average, GRAPES disconnects 12 candidate graphs (Figure S4) into about 12 smaller connected components. GRAPES consumes more memory than VF2 and GGSX and GRAPES has the same filtering power as GGSX (Figure S4). GRAPES indexing allows parallelism and fast matching phases resulting in a faster tool.

Concerning scalability, GRAPES scales well on both Building and Matching phase in all datasets (Figure S5). However, scalability is better when the data has complex structure and queries are time consuming such as in the *PCM* and *PPI* datasets.

Finally we present the performance of the tools when the queries have no matches (Figures S6, S7 S8, and S9). This may result from topological or label differences between the query and the data graphs. Building time and memory consumption do not change. The number of matching graphs is 0 for all datasets. GRAPES generates less candidates in all datasets. GRAPES generates 522.67 (1,563.88) components in *AIDS*, 28.33 (41.30) in *PDBS*, 7.42 (9.07) in *PCM*, and 5.57 (3.73) in *PPI*. Since the queries are not present in the datasets, features of the queries are rarely present in the graphs. As a consequence, only few candidates are generated together with a small number of connected components. Concerning matching time, GRAPES is the fastest tool in all datasets but the simplest structured one (i.e., *AIDS*).

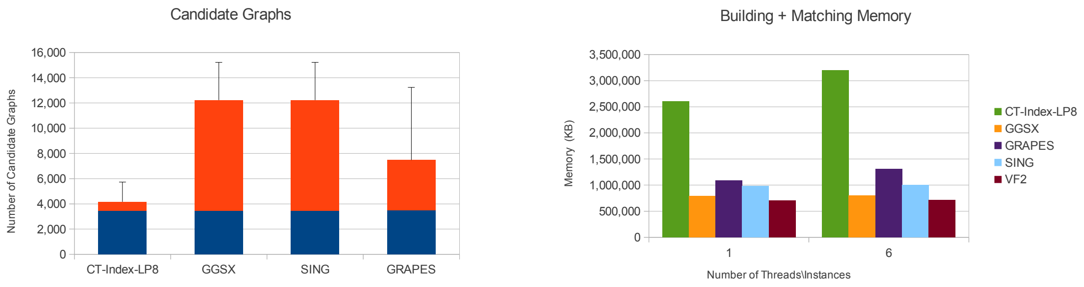

Figure S1. Number of candidate graphs and memory consumption of the compared tools in the building and matching phases on the *AIDS* dataset.

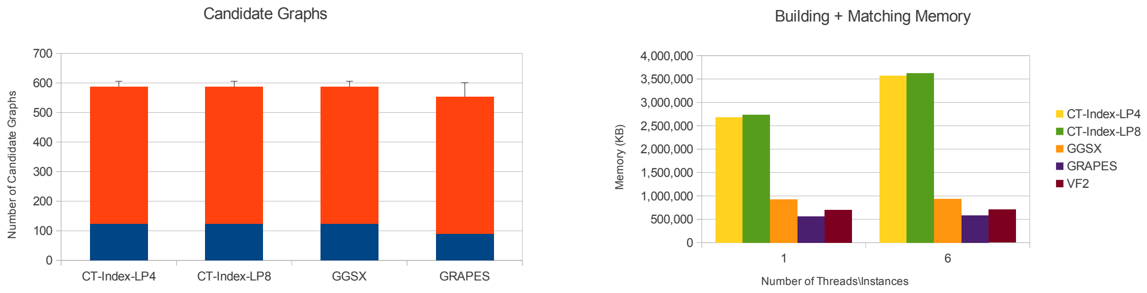

Figure S2. Number of candidate graphs and memory consumption of the compared tools in the building and matching phases on the *PDBS* dataset.

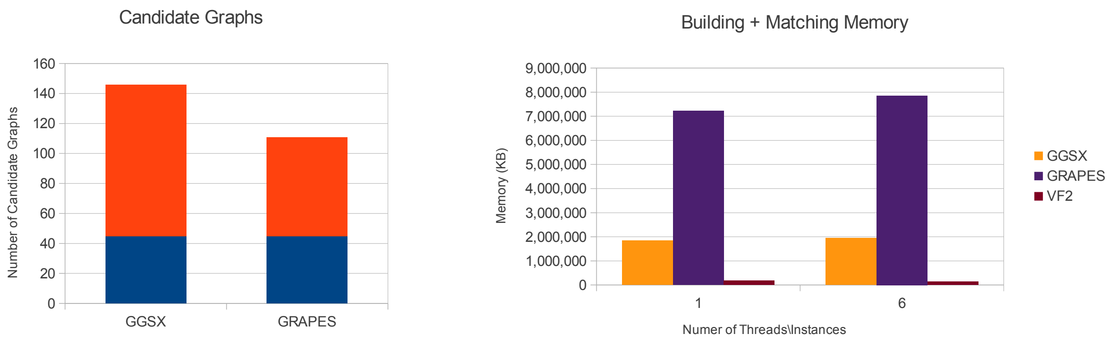

Figure S3. Number of candidate graphs and memory consumption of the compared tools in the building and matching phases on the *PCM* dataset.

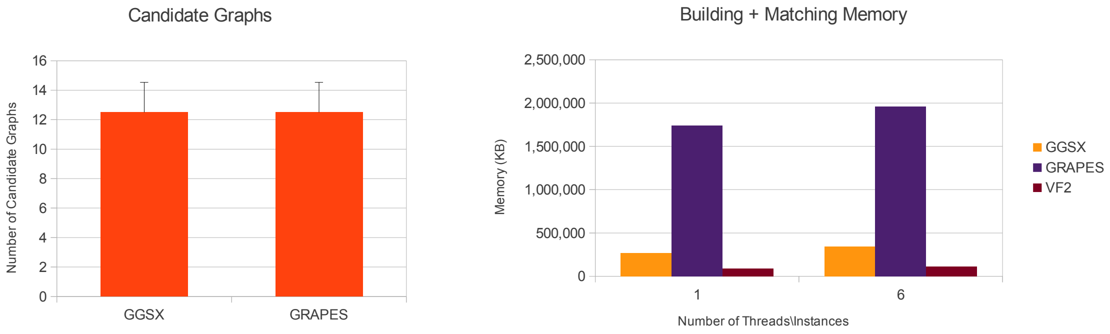

Figure S4. Number of candidate graphs and memory consumption of the compared tools in the building and matching phases on the *PPI* dataset.

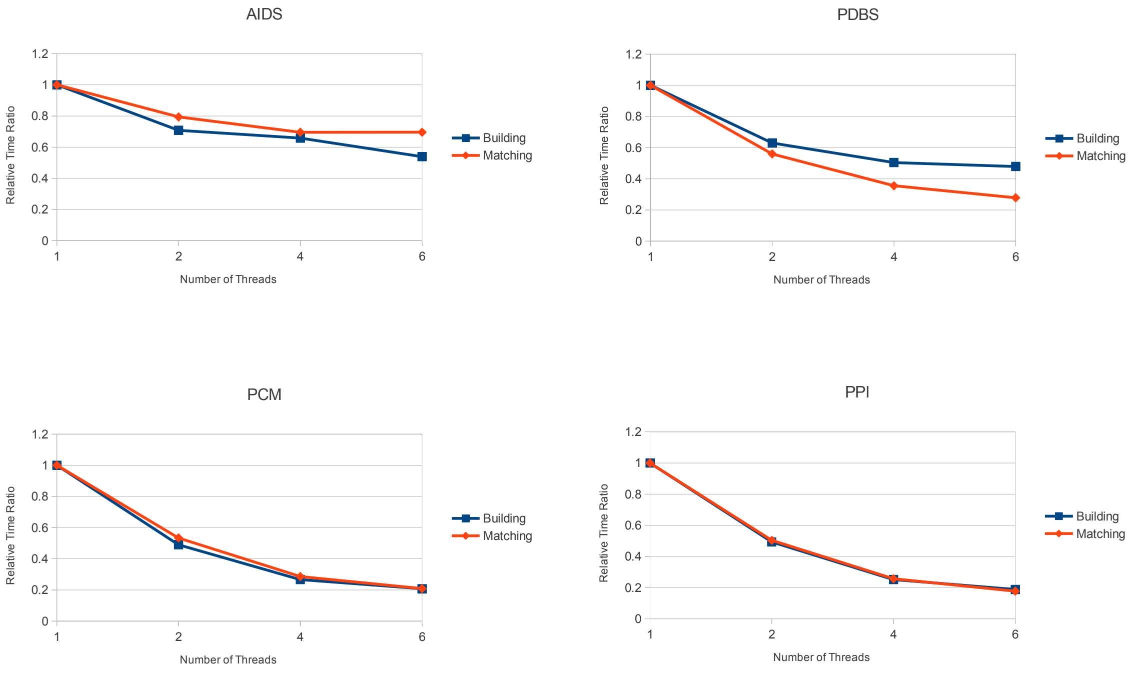

Figure S5. GRAPES scalability on the *AIDS*, *PDBS*, *PCM* and *PPI* datasets.

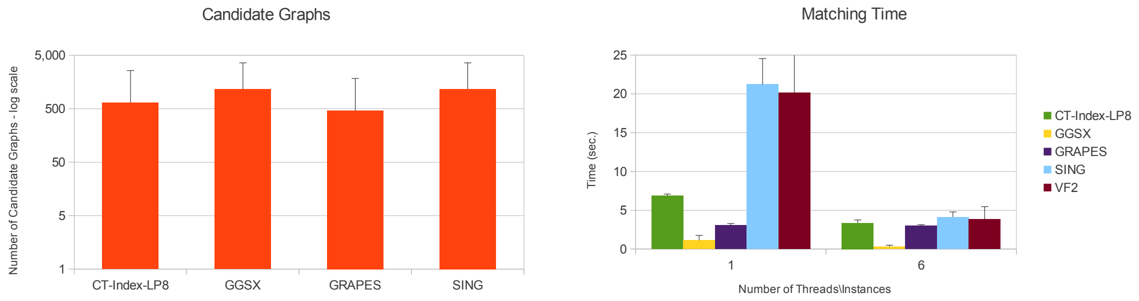

Figure S6. Behavior of the compared tools on the *AIDS* dataset when queries are not present in the dataset.

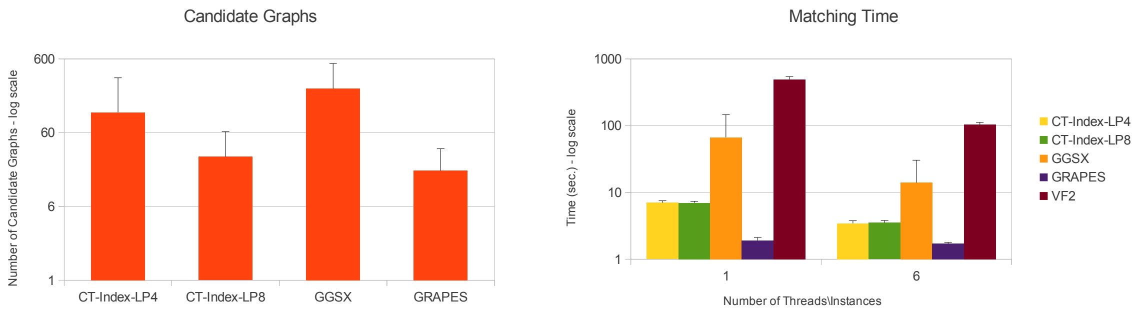

Figure S7. Behavior of the compared tools on the *PDBS* dataset when queries are not present in the dataset.

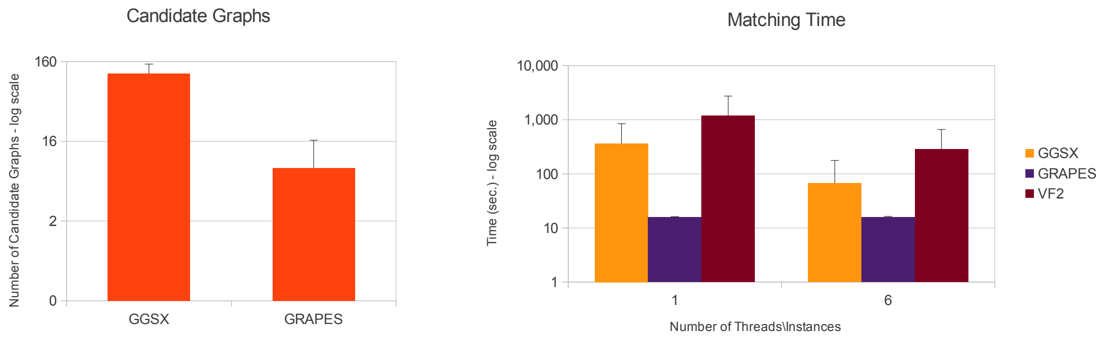

Figure S8. Behavior of the compared tools on the *PCM* dataset when queries are not present in the dataset.

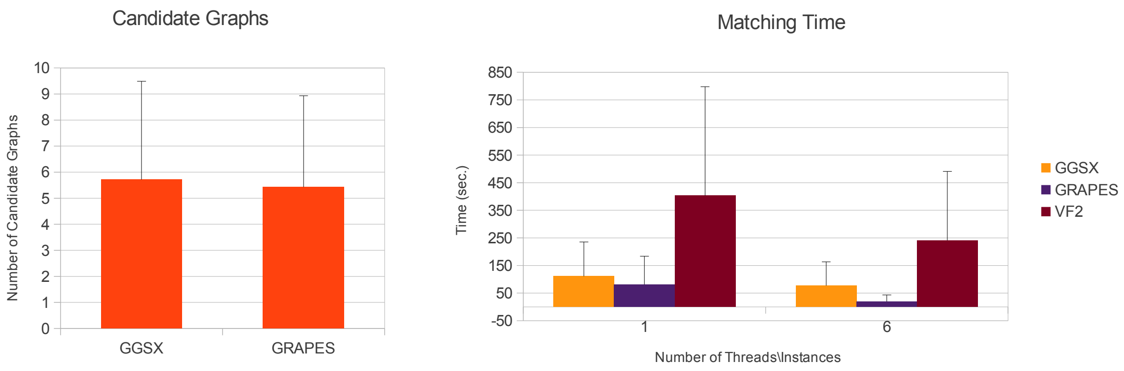

Figure S9. Behavior of the compared tools on the *PPI* dataset when queries are not present in the dataset.
