# Supplementary material for: GRAPES: A Software for Parallel Searching on Biological Graphs Targeting Multi-Core Architectures
Source: PLoS One. 2013 Oct 22;8(10):e76911. doi: 10.1371/journal.pone.0076911 (PMC3805575; doi:10.1371/journal.pone.0076911)
Supplement: File S2 — GRAPES user manual. (PDF) [file pone.0076911.s002.pdf]

GRAPES v 2.9.mp.vf2

User's Guide

developed by

Rosalba Giugno[1], Vincenzo Bonnici[2], Nicola Bombieri[2], Alfredo Pulvirenti[1], Alfredo Ferro[1] and Dennis Shasha[3]
University of Catania[1], University of Verona[2] and New York University[3]

GRAPES - Version 2.9

GRAPES is a querying system for parallel searching in databases of graphs, and single target graph, using symmetric multiprocessing (SMP) architectures. It implements a parallel version of well established graph searching algorithms providing efficient solutions for graphs indexing and matching.

GRAPES is developed in C++ under GNU\Linux using POSIX Threads programming and no further dependencies out of standard GNU C++ library. It can also be compiled to run under Windows and MAC OSx systems.


```
Copyright (c) 2013 by Rosalba Giugno

GRAPES is provided under the terms of The MIT License (MIT):

Permission is hereby granted, free of charge, to any person
obtaining a copy of this software and associated
documentation files (the "Software"), to deal in the
Software without restriction, including without limitation
the rights to use, copy, modify, merge, publish, distribute,
sublicense, and/or sell copies of the Software, and to
permit persons to whom the Software is furnished to do so,
subject to the following conditions:

The above copyright notice and this permission notice shall
be included in all copies or substantial portions of the
Software.

THE SOFTWARE IS PROVIDED "AS IS", WITHOUT WARRANTY OF ANY
KIND, EXPRESS OR IMPLIED, INCLUDING BUT NOT LIMITED TO THE
WARRANTIES OF MERCHANTABILITY, FITNESS FOR A PARTICULAR
PURPOSE AND NONINFRINGEMENT. IN NO EVENT SHALL THE AUTHORS
OR COPYRIGHT HOLDERS BE LIABLE FOR ANY CLAIM, DAMAGES OR
OTHER LIABILITY, WHETHER IN AN ACTION OF CONTRACT, TORT OR
OTHERWISE, ARISING FROM, OUT OF OR IN CONNECTION WITH THE
SOFTWARE OR THE USE OR OTHER DEALINGS IN THE SOFTWARE.
```


GRAPES is distributed under the MIT license. This means that it is free for both academic and commercial use. Note however that some third party components in **GRAPES** require that you reference certain works in scientific publications. You are free to link or use **GRAPES** inside source code of your own program. If do so, please reference (cite) **GRAPES** and this website. We appreciate bug fixes and would be happy to collaborate for improvements.

Usage

GRAPES is developed in C++ under GNU\Linux using POSIX Threads programming and no further dependencies out of standard GNU C++ library. It works on Unix and Mac OS X systems with G++ installed, and it can be compiled under Windows using Gygwin.

Build Source Code

Executables are available only after building source code on your system.

```
cd GRAPES-2.9.mp.vf2
make clean
make -B
```

At the end of the build process,the command grapes is available.

Database Index Construction

Build the index of the given database of graphs.

```
./grapes NTHREADS -b -[gfu|gfd] db_file [-lp lp]
```

Command parameters:

`NTHREADS`	number of parallel threads	
`-[gfu|gfd]`	input file format	
	`-gfu`	undirected graphs file format
	`-gfd`	directed graphs file format
`db_file`	textual graphs database file. All database graphs are stored into the same file.	
`[-lp lp]`	OPTIONAL, specify feature paths length, namely the depth of the DFS which extract paths. `lp` must be greater than 1, eg `-lp 3`. Default value `-lp 4`.	

The indexing phase ever produces the `db_file.index.grapes` file in which the database index is stored.

Querying

Querying operations are available by the command `grapes` that allow to search a single pattern graph (query) inside the database of graphs.

Restrictions:

- the system allows to search only for sub-isomorphisms between the query (pattern graph) and the database
- one query per time can be processed, thus the input query file cannot contains more than one graph
- before run a query, the database index must have been computed by the command `grapes -b` and the resultant `.index.grapes` file must be maintained in the same directory of the database textual file.

Search by coarse-grained matching phase.

```
./grapes NTHREADS -f -[gfu|gfd] db_file query_file -[no|console|file] [-lp lp]
```

Parameters:

`NTHREADS`	number of parallel threads	
`-[gfu|gfd]`	input file format	
	`-gfu`	undirected graphs file format
	`-gfd`	directed graphs file format
`db_file`	textual graphs database file	
`query_file`	textual query graph file. It must contain just one graph.	
`-[no|console|file]`	print found matches (sub-isomorphisms)	
	`-no`	do not print
	`-console`	print matches on screen
	`-file`	print matches on files. Each thread prints found matches in a different file
`[-lp lp]`	OPTIONAL ONLY IF the database index was built with a `lp` value different from 4, specify feature paths length, namely the depth of the DFS which extract paths. `lp` must be greater than 1, eg `-lp 3`. Default value `-lp 4`.	

Input Formats

Graphs are stored in text files containing one or more items. The current input format allows the description of undirected `-gfu` or directed `-gfd` graphs with labels on nodes.

Graph description language format:

```
#[graph_name]
[number of nodes]
[label_of_first_node]
[label_of_second_node]
...
[label_of_last_node]
[number of edges]
[node id] [node id]
[node id] [node id]
...
```

GRAPES assigns IDs to nodes following the order in which they are written in the input file, starting from 0.

Restrictions:

- multigraphs and hypergraphs are not allowed
- attributes (labels) can be assigned only to the nodes (vertices) of the graph
- labels are case-sensitive
- `[graph_name]` and labels can not contain blank characters (space, tabs and so on)
- duplicated edges are ignored
